# Supplementary material for: GLIS1, Correlated with Immune Infiltrates, Is a Potential Prognostic Biomarker in Prostate Cancer
Source: Int J Mol Sci. 2023 Dec 29;25(1):489. doi: 10.3390/ijms25010489 (PMC10779070; doi:10.3390/ijms25010489)
Supplement: Supplementary file 1 [file ijms-25-00489-s001.zip › ijms-2743417-supplementary.pdf]

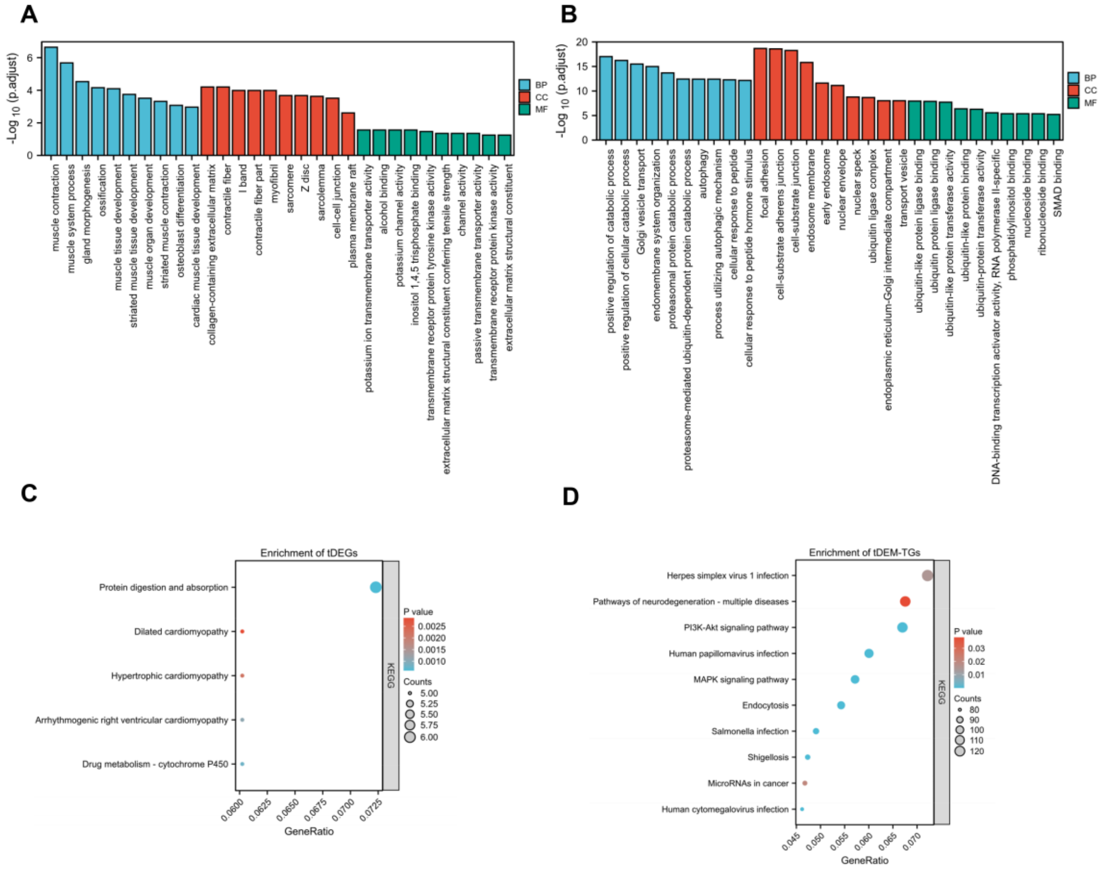

Figure S1. The functional and pathway enrichment analyses of tDEGs and tDEM-TGs. A, Biological process (BP), cellular component (CC) and molecular function (MF) analyses for tDEGs, the top 10 for each GO classification were listed. B, BP, CC and MF analyses for tDEM-TGs, the top 10 for each GO classification were listed. C, KEGG pathway enrichment analysis for tDEGs. D, KEGG pathway enrichment analysis for tDEM-TGs, the top 10 were listed.

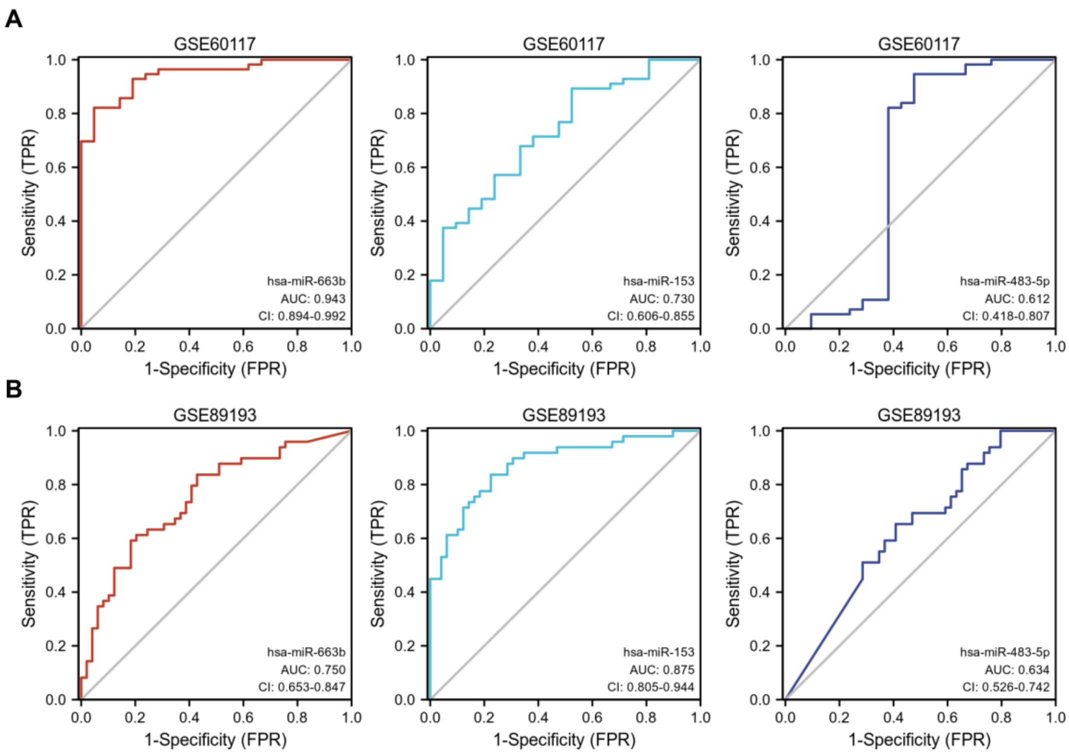

Figure S2. The diagnostic potential of key miRNAs. ROC curves of key miRNAs (hsa-miR-663b, hsa-miR-153, hsa-miR-483-5p) in GSE60117 (A) and GSE89193 (B) datasets.

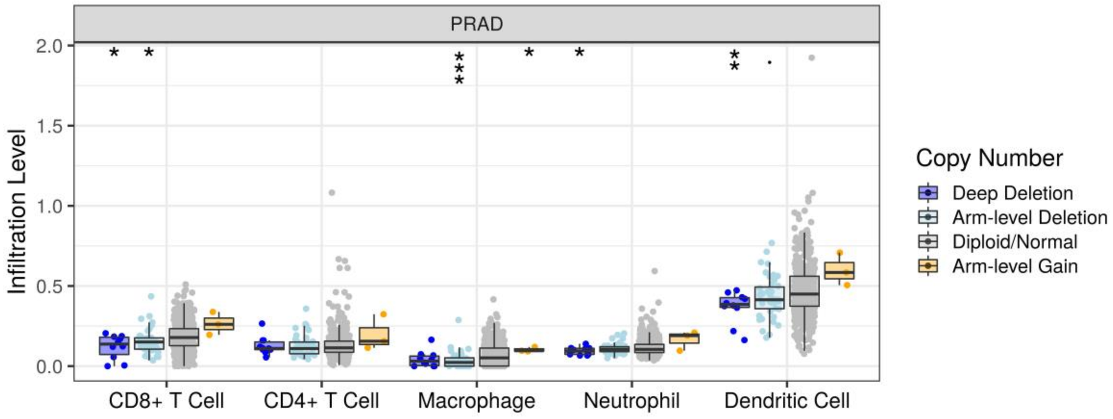

Figure S3. Correlation between tumor infiltration levels and different somatic copy number alterations (SCNAs) of GLIS1 in prostate adenocarcinoma (PRAD). The deep deletion of GLIS1 was significantly associated with decreased tumor infiltration levels in PRAD. In addition, arm-level deletion of GLIS1 was significantly associated with decreased CD8+ T cells, macrophage and dendritic cells infiltration levels. * p < 0.05, ** p < 0.01, *** p < 0.001.
